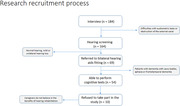# Challenges engaging patients with dementia and their care partners in hearing rehabilitation research

**DOI:** 10.1002/alz.086736

**Published:** 2025-01-09

**Authors:** Luciana Macedo de Resende, Mariane Gomes Machado, Thaís Helena Machado, Michele Gomes Ferreira, Douglas Thuller, Paulo Caramelli

**Affiliations:** ^1^ Universidade Federal de Minas Gerais, Belo Horizonte, Minas Gerais Brazil; ^2^ Federal University of Minas Gerais, Belo Horizonte Brazil

## Abstract

**Background:**

Hearing rehabilitation has been a promising approach to improve cognitive outcomes. An ongoing study identified some barriers to engage patients in counseling sessions and using their hearing devices. Here we present the results from the first stage of a Sense‐Cog Brazil pilot study, the recruitment phase.

**Method:**

Observational pilot study with 184 patients with dementia assisted in a Neurocognitive Outpatient Clinic from the University Hospital (Hospital das Clínicas/ Universidade Federal de Minas Gerais, Belo Horizonte, Brazil). Procedures in the two‐step recruitment phase were, first performing an audiometric screening, and second a thorough neurocognitive testing and complete audiological assessment. Inclusion criteria were as follows: bilateral sensorineural hearing loss, diagnosis of dementia, availability for patient and communicative partner to participate in hearing rehabilitation and counseling groups. Patients with dementia with Lewy bodies, aphasia or frontotemporal dementia were excluded from the pilot. Descriptive analysis was performed.

**Result:**

From the initial 184 patients, 20 (10.9%) were not able to perform hearing screening due to difficulties with audiometric tasks or obstruction of the external ear canal. A total of 164 (89.1%) patients were able to finalize audiometric screening and 69 (42.7%) were referred to bilateral hearing aids fitting. However only 54 (32.9%) were able to perform cognitive tests. From these 54 patients included in the pilot, 10 (18.5%) refused to take part in the study. Main reasons were: caregivers not believing in hearing rehabilitation benefits, not having time to participate, and not acknowledging hearing results. Also, some difficulties in performing the hearing assessment, a test which requires an experienced audiologist, the patient’s cooperation and comprehension of the task.

**Conclusion:**

The patients’ loss in the recruiting and selecting process was mainly due to the lack of perception of the possible benefits of hearing rehabilitation, especially from the caregivers' perspective. This is an important issue to be addressed in the continuation of the study. An additional challenge was having a trained team to assess dementia patient’s hearing. Furthermore, special care should be taken when assessing cognition with dementia patients with hearing loss.